# Lupeol Acetate and α-Amyrin Terpenes Activity against *Trypanosoma cruzi*: Insights into Toxicity and Potential Mechanisms of Action

**DOI:** 10.3390/tropicalmed8050263

**Published:** 2023-05-03

**Authors:** Daniel Pardo-Rodriguez, Andres Cifuentes-López, Juan Bravo-Espejo, Ibeth Romero, Jorge Robles, Claudia Cuervo, Sol M. Mejía, Jair Tellez

**Affiliations:** 1 Grupo de Enfermedades Infecciosas, Pontificia Universidad Javeriana, Bogotá 110231, Colombia; 2 Grupo de Investigación Fitoquímica Universidad Javeriana (GIFUJ), Pontificia Universidad Javeriana, Bogotá 110231, Colombia; 3 Grupo de Productos Naturales, Universidad del Tolima, Tolima 730006299, Colombia; 4 Department of Chemistry, Rutgers University, Newark, NJ 07102, USA; 5 Escuela de Pregrados, Dirección Académica, Vicerrectoría de Sede, Universidad Nacional de Colombia, Sede, De La Paz 202010, Colombia

**Keywords:** Chagas disease, terpenes trypanocidal effect, cysteine synthase, docking, molecular dynamics, *Trypanosoma cruzi*

## Abstract

Background: Chagas disease is a potentially fatal disease caused by the parasite *Trypanosoma cruzi*. There is growing scientific interest in finding new and better therapeutic alternatives for this disease’s treatment. Methods: A total of 81 terpene compounds with potential trypanocidal activity were screened and found to have potential *T. cruzi* cysteine synthase (TcCS) inhibition using molecular docking, molecular dynamics, ADME and PAIN property analyses and in vitro susceptibility assays. Results: Molecular docking analyses revealed energy ranges from −10.5 to −4.9 kcal/mol in the 81 tested compounds, where pentacyclic triterpenes were the best. Six compounds were selected to assess the stability of the TcCS–ligand complexes, of which lupeol acetate (ACLUPE) and α-amyrin (AMIR) exhibited the highest stability during 200 ns of molecular dynamics analysis. Such stability was primarily due to their hydrophobic interactions with the amino acids located in the enzyme’s active site. In addition, ACLUPE and AMIR exhibited lipophilic characteristics, low intestinal absorption and no structural interferences or toxicity. Finally, selective index for ACLUPE was >5.94, with moderate potency in the trypomastigote stage (EC_50_ = 15.82 ± 3.7 μg/mL). AMIR’s selective index was >9.36 and it was moderately potent in the amastigote stage (IC_50_ = 9.08 ± 23.85 μg/mL). Conclusions: The present study proposes a rational approach for exploring lupeol acetate and α-amyrin terpene compounds to design new drugs candidates for Chagas disease.

## 1. Introduction

Chagas disease (ChD) is an infection caused by the protozoan *Trypanosoma cruzi*, which is naturally distributed from southern United States to Argentina, largely in Latin America, and is transmitted to humans from wild animals, mainly insects of the Triatominae family [1]. Two drugs are currently available for ChD treatment, Benznidazole (LAFEPE, Recife, Brazil; ELEA, Buenos Aires, Argentina) and Nifurtimox (Bayer, Buenos Aires, Argentina). Both drugs are nitroheterocyclic compounds that are widely used during the acute and congenital phases of *T. cruzi* infection. However, the efficacy of the treatment during the chronic phase remains debatable [2,3]. In addition, these nitroheterocyclic compounds have been reported to have marked side effects, and systemic toxicity associated with long treatment times and high doses [4,5]. Due to the limited number of therapeutics available for ChD treatment, new trypanocidal alternative drugs are urgently needed. In this respect, other therapeutic targets are especially attractive for development as selective inhibitors of essential parasitic metabolic pathways for ChD therapy. However, this approach has rarely been explored in the context of neglected tropical diseases.

Cysteine synthase (CS) in trypanosomatids, such as *T. cruzi* and *Leishmania* (*L.*) spp., should be explored as a potential drug target since it plays a key role in the biosynthesis of cysteine, an important amino acid that comprises part of the central block of trypanothione, a thiol molecule essential for the redox balance in trypanosomatids [6]. CS presents significant differences at the biochemical and structural levels, with its closest homolog in humans being cystathionine β-synthase [7]. In addition, CS has been correlated with oxidative stress survival in *T. cruzi* and the antimonial response in *Leishmania braziliensis* [8]. A correlation between increased expression levels of CS and heightened resistance to antimonial compounds in *L. braziliensis* was revealed. The protective association between higher CS expression and activity and parasite survival under stress conditions was demonstrated through the enhanced ability of *L. braziliensis* overexpressing CS to withstand oxidative stress in vitro induced by hydrogen peroxide (H_2_O_2_), as well as antimonial trivalent (SbIII) and pentavalent (SbV) compounds, in comparison to the wild-type *L. braziliensis*. Additionally, elevated expression of CS resulted in decreased susceptibility to antimony drugs [8]. Due to the critical role of cysteine in growth, pathogenicity, protection against redox damage, drug resistance across various pathogens, and the absence of the CS enzyme in mammals, CS proteins have been suggested as promising targets for the development of novel anti-microbial drugs [9].

The terpenoids group has attracted attention due to its remarkable biological activities, such as cardiovascular effects [10], anti-carcinogenic [11], anti-inflammatory [12,13], antiviral and antiparasitic [14]. In fact, some of these terpenoids are already commercially distributed for the treatment of different pathologies, such as artemisinin, a sesquiterpene lactone peroxide originally isolated from *Artemisia annua* used as an antimalarial [15] and the cyclic diterpene paclitaxel derived from the taxane nucleus obtained from *Taxus brevifolia* used as a treatment for cancer [16]. Thus, the biological potential and low toxicity, in both in vivo and in vitro models, have made this group of molecules an important source of bioactive compounds and chemical skeletons for their derivatization [17].

Despite the fact that triterpenes have been widely described as trypanocidal agents [18,19,20,21,22], their mode of action has not been completely elucidated. Recently, two in silico studies reported the results of molecular docking between many terpenoids and certain targets of importance in regard to infectious diseases. The docking of a group of isoprenoids to the active sites of 29 proteins of Leishmania major, Leishmania donovani, Leishmania mexicana and Leishmania infantum and the docking of some monoterpenoids to enzymes, such as nicotinamidase, uridine diphosphate-glucose pyrophosphorylase and methionyl t-RNA synthetase were demonstrated. In addition, germacranolide sesquiterpenoids exhibited affinity for methionyl t-RNA synthetase and dihydroorotate dehydrogenase [23]. On the other hand, Coy and Bernal (2014) also evaluated the interactions between sesquiterpenoids and four drug–enzymatic targets, pteridine reductase-1, N-myristoyl transferase, cysteine synthase and trypanothione synthetase. Two sesquiterpenic coumarins showed inhibitory activity against pteridine reductase and trypanothione synthetase, while some xanthanolides exhibited an enhanced affinity for CS [24]. The present study aimed to assess the affinities and stability of the complex formed between terpenic compounds and the CS enzyme of *T. cruzi*, as well as to evaluate the in vitro trypanocidal capacities of the promising compounds of the in silico analysis. Thus, it seeks to rationalize the exploration of anti-trypanosome molecules as alternative therapies for the treatment of ChD.

## 2. Materials and Methods

### 2.1. Pairwise Sequence Alignment and Preparation of the 3D T. cruzi CS Enzyme Model

To evaluate the conservation of TcCS and LmCS, the amino acid sequences were compared using the pairwise sequence alignment tool from the EMBL-EBI server [25]. Both CS sequences were obtained from the UniProtDB platform (LmCS: Q4Q159, TcCS: Q4CST7). The alignment was outlined using the ESPript 3 tool (http://espript.ibcp.fr/) [26], where the active site residues of LmCS reported in the PDB database were manually specified. To elucidate the tertiary structure of the *T. cruzi* CS (TcCS) protein, a homology model was generated using the structure of the CS enzyme from *Leishmania major* (LmCS, PDB code: 4AIR) as a template using the HHpred algorithm on the MODELLER server (https://toolkit.tuebingen.mpg.de/tools/hhpred, accessed on 20 June 2020). The X-ray crystallographic structure of LmCS was obtained from the Protein Data Bank (PDB) at a resolution of <2.4 Å, and LmCS (PDB code: 4AIR) was used as a template for TcCS model construction. Validation of each protein model was carried out via the construction of Ramachandran plots and Qualitative Model Energy ANalysis (QMEAN) using the Structure Assessment tool on the SWISS-MODEL server (https://swissmodel.expasy.org/assess/, accessed on 20 June 2020). Additionally, the structural comparison was conducted using RMSD values between the models generated using homology (TcCS), the crystallographic structure used as template (LmCS) and the model generated using the AlphaFold server.

In the protein structure, the nonessential water molecules, ions and ligands were removed from the TcCS protein model, and polar hydrogens and standard protonation states were assigned using AutoDockTools (ADT) v1.118 [27] and Maestro [28]. The active site of TcCS was constructed based on comparisons with the residues known to stabilize the cofactor pyridoxal phosphate (PLP) in TcCS, which included Lys51, Asn82, Ser274 and Gly186 [29,30].

### 2.2. Ligand Preparation

The structures of eighty-one terpenic compounds, including a known CS inhibitor (PUBCHEM: 247228), O-acetyl-DL-serine (OAS) (natural substrate), and a cofactor (PLP) were downloaded from ZINC (http://zinc.docking.org/, accessed on 20 June 2020) and PUBCHEM (https://pubchem.ncbi.nlm.nih.gov/, accessed on 20 June 2020) databases. The selected terpenes corresponded to molecules with potential antitrypanosomal and/or antileishmanial activity and were classified according to their carbon number as monoterpenes (C_10_), sesquiterpenes (C_15_), diterpenes (C_20_), sesterterpenes (C_25_), triterpenes (C_30_) and terpenic coumarins, terpenic alkaloids and saponins. Compound geometry was optimized through a random conformational search for at least 100 conformers by employing Universal Force Field in the Avogadro software [31]. The energetically favored conformer of every compound was selected as the initial ligand structure for use in further analyses (Table 1).

### 2.3. Molecular Docking

The optimal energy conformations for the ligands interacting with the TcCS and LmCS protein active sites were analyzed using Autodock vina v1.1.2; all default docking parameters were utilized, except the number of binding poses that was fixed to twenty for each ligand, as described previously [32]. During the analysis, the active site of each CS protein was treated as a rigid molecule, whereas the ligands were treated as flexible molecules. The active site was delimited within a cubic box that was 30 Å in size. Interactions between the ligands and the proteins with the highest binding affinity were assessed using the Protein–Ligand Interaction Profiler server (https://projects.biotec.tu-dresden.de/plip-web/plip) [33].

### 2.4. Molecular Dynamics Simulations

The protein–ligand complex structure for each molecular dynamic (MD) simulation was built based on the best pose score obtained from the molecular docking analysis. To perform each MD simulation, the topology files for each ligand structure were generated with SwissParam [34]. MD simulation was performed using the Gromacs 2018.8 package and the CHARMM27 force field [35]. First, the protein–ligand complexes were prepared based on energy minimization in water, using the steepest descent energy value and a TIP3P water molecule model to center each system in a cubic box of specific vectors [36]. Then, the MD equilibration of an isochoric–isothermal ensemble (NVT) at 2 ns followed by that of an isothermal–isobaric ensemble (NPT) at 2 ns was performed. The neutralization of each protein–ligand complex was carried out by adding six Na^+^ counterions to the continuous solvent phase [37]. This neutralization took place due to the total negative charge of each protein–ligand complex as a product of the sum of the all charged protein amino acids under neutral pH conditions. Energy minimization was achieved when the system did not exceed a tolerance of 10 kJ/mol, and bonds were subjected to holonomic constraints by employing the LINCS algorithm [38,39]. In addition, the modified Berendsen coupling V-rescale algorithm in the NVT ensemble was used to control the temperature of the complexes [40]. The NPT ensemble was constructed using the Parrinello–Rahman coupling algorithm at 1 atm for pressure control [41]. To generate a 200 ns MD simulation of the protein, each protein–ligand complex simulation was performed using periodic boundary conditions at 310 K and 1 atm with a 1.2 nm Verlet cutoff scheme for the short-range van der Waals cutoff [42]. For each protein–ligand complex, root mean square deviation (RMSD), root mean square fluctuation (RMSF), and hydrogen bond analyses were performed using the Gromacs software package and Visual Molecular Dynamics (VMD) v 1.9.4 [43].

### 2.5. ADME and PAIN Predictions

The terpenes identified as potential TcCS inhibitors were subjected to the absorption, distribution, metabolism, excretion (ADME) and pan-assay interference compound (PAIN) analyses using the SwissADME and ADMETlab tools, as previously described [44,45].

### 2.6. Activity against T. cruzi Extracellular Forms

Trypomastigotes (5 × 10^5^ parasites/well) of Y-strain (MHOM/BR/00/Y), discrete typing unit II (TcII), were seeded in 96-well plates and incubated for 24 h with decreasing concentrations of lupeol acetate and α-amyrin (MedChemExpress, Monmouth Junction, NJ, USA) from 94 μg/mL to 3 μg/mL. As negative control, parasite with medium alone and parasites with 1% DMSO were used and as positive control, Nifurtimox (NFX) (Sigma-Aldrich, St. Louis, MO, USA) at 2.5 μg/mL was employed. Treatment effect on trypomastigote viability was determined by hemocytometer count [46]. The concentration that eliminated 50% of the trypomastigote population (EC_50_) were calculated using r studio software employing a probit analysis. All assays were performed in triplicate and three independent biological replicates were carried out.

### 2.7. Activity against Intracellular Forms of T. cruzi

A total of 1 × 10^5^ VERO cells (Green Monkey renal fibroblast-like cells (ATCC CCL-81, Manassas, VA, USA) were cultured in 6-well plates for 12 h. In the subsequent step, cells were infected with *T. cruzi* trypomastigotes at a 1:10 (cell:parasite) ratio. After 12 h of infection, uninternalized trypomastigotes were eliminated by washing the cultures. The cells were then incubated with the same concentrations of lupeol acetate and α-amyrin (MedChemExpress, Monmouth Junction, NJ, USA) used in the previous assay, for 48 h at 37 °C and 5% CO_2_. Lastly, the cultures were washed with PBS (Eurobio), fixed with methanol and stained with Giemsa stain (Sigma-Aldrich). To determine the extract activity, the percentage of infected cells and the number of amastigotes per infected cell in both treated and untreated cultures (association index) were calculated. This was conducted by counting 200 randomly distributed cells under a light microscope at a 100× magnification [47]. Using R Studio software with a probit analysis, the parasitic population (IC_50_) was calculated by comparing the association indices of treated and untreated parasites. This type of experiment was performed in triplicate, and three independent biological replicates were carried out to ensure the reliability and reproducibility of the results.

### 2.8. Cytotoxic Activity on VERO Cells

VERO cells were seeded at a density of 5 × 10^3^ cells/well in 96-well plates and incubated at 37 °C and 5% CO_2_ for 48 h with the same concentrations of lupeol acetate and α-amyrin (MedChemExpress, NJ, USA) from the parasite’s assays. MTT colorimetric assay was used to estimate the cytotoxic effect. The cytotoxicity of the compounds was assessed by determining the concentration that caused 50% reduction in cell viability (CC_50_), as previously described. Triplicate assays were performed, and three independent biological replicates were carried out. The selectivity of the compounds was evaluated by calculating the selectivity index (SI) ratio between CC_50_ in VERO cells and EC_50_ (trypomastigote) or IC_50_ (amastigote) in *T. cruzi* stages [48].

## 3. Results and Discussion

### 3.1. Pairwise Sequence Alignment and Assessment of Model Quality

**Figure 1:**
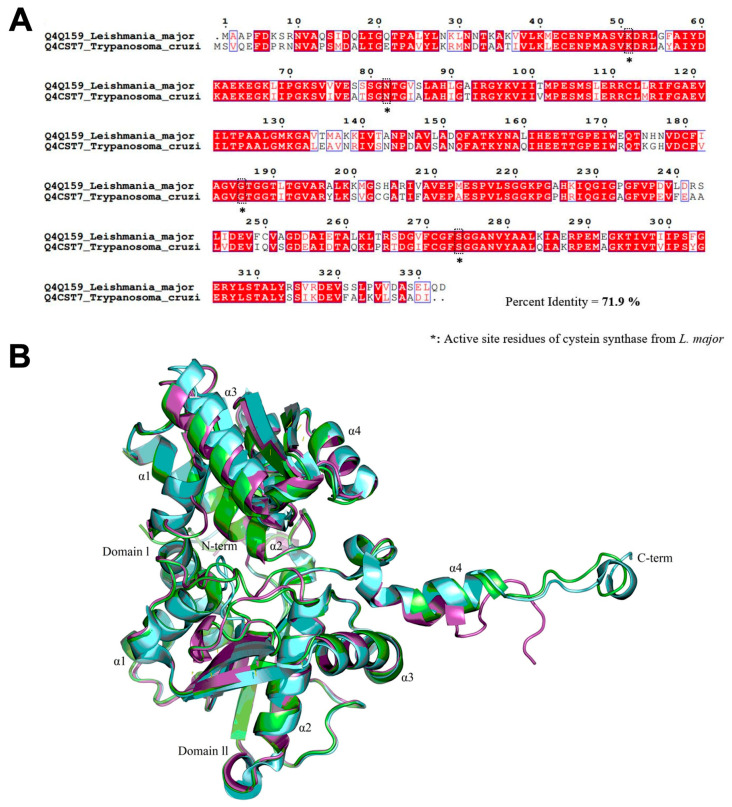
General structure of TcCS and alignment of its sequence with a homologous protein. (**A**) Sequence alignment of TcCS with LmCS. (**B**) Representation of the general structure of the protein modeled by homology, TcCS model by homology (magenta), TcCS AlphaFold model (green) and the crystallographic protein LmCS (cyan).

Pairwise sequence alignment of LmCS and TcCS revealed that 71.9% of the TcCS residues are homologous (both position and type) to those of LmCS (residues colored in red, Figure 1A). Additionally, some changes were observed in residues with physical and chemical similarities (residues demarcated with the blue squares). These residues are not considered to significantly influence the folding or function of the enzymes because they do not reside in the reported LmCS active site [29]. Finally, the sequence alignment indicated that the residues involved in stabilizing PLP as a cofactor of TcCS were conserved with respect to those reported in LmCS (residues marked with black dotted lines) (Figure 1A).

The modeled enzyme is a monomer comprising two domains: domain I, which mainly forms a four-stranded β sheet surrounded by four α helices, and domain II, made up of four α helices and six β sheets (Figure 1B). Alignment of the tertiary structures of the model protein TcCS (Figure 1B, magenta) and the crystallographic protein LmCS (Figure 1B, cyan) revealed the placement of most of the ordered protein structures, except for the four α helices of domain II adjacent to the C-terminus, in which some decoupling was observed. On the other hand, a loop surface comprising residues 214 to 241 was disordered, and thus excluded from the crystallographic model, thereby preventing correct alignment with the model generated using homology analysis. Finally, the model generated using homology was compared against the crystallographic structure obtained from the PDB and the model generated in AlphaFold available in the UniProt database, finding protein structure conservation with low RMSD values of 2761 and 2310 Å, respectively.

The QMEAN scoring function estimates both global and local quality according to the superpositions of the colored models to the predicted residual errors, ranging from blue to red. The colored regions of the red spectrum correspond to residues that deviate from the native conformation [49]. This scoring function showed that both the TcCS and LmCS models had good stereochemical quality (residues colored in blue, supporting information Figures S1 and S2) except for the C-terminal region of TcCS, which had low quality. However, this zone does not influence the active site reported for its homologous protein LmCS.

### 3.2. Molecular Docking of Terpenes

The molecular docking results of the terpenes screened against the TcCS and LmCS proteins showed different docking scores (Table 2). The terpenic compounds were categorized based on the number of carbon atoms as monoterpenes, sesquiterpenes, diterpenes, sesterterpenes, tetracyclic triterpenes, pentacyclic triterpenes and terpenic coumarins. The other three compounds were used as controls to compare the binding energies of these molecules to the values obtained for the terpenic compounds against the TcCS and LmCS proteins. The results of molecular docking against the TcCS protein showed binding energy values ranging from −4.9 to −10.3 kcal/mol and LmCS protein values ranging from −5.4 to −12.2 kcal/mol.

Regarding the evaluated compounds, monoterpenes, diterpenes, sesquiterpenes, and sesterterpenes had the lowest affinities for TcCS and LmCS. On the other hand, the compounds belonging to the group of pentacyclic triterpenes had the highest affinities for TcCS and LmCS. Other compounds, such as terpenic coumarins, exhibited more affinity for LmCS than for TcCS. Overall, 21.0% (17/81) of the pentacyclic terpenes exhibited higher affinities for binding to the TcCS protein than to the natural substrate and 33.3% (27/81) to NSC61610, a known inhibitor of OAS, and 33.3% (27/81) of the compounds presented higher binding affinities for the LmCS protein and were thus classified as potential inhibitors of each CS enzyme. Pentacyclic terpenes promising compounds, especially of plant origin, have been previously reported as possible drugs for parasitic diseases, however, their potential as alternative and their mode of action remain unclear [14].

The docking energies of the grouped terpenic compounds showed that the pentacyclic triterpene molecules exhibited the highest mean docking energies for the TcCS protein; among them, 3-O-caffeicoleanolic acid, 20-α-hydroxytingenone, enoxolone and 3-O-trans-caffeoyltormentic acid showed the highest docking energy values of −10.3, −10.1, −10.1 and −10.0 kcal/mol, respectively (Table 3). Additionally, these triterpenes exhibited better coupling energies than the supported inhibitor NSC61610, which had a docking energy of −9.6 kcal/mol for TcCS.

### 3.3. Analysis of the Molecular Interactions between Pentacyclic Triterpenes and the TcCS Enzyme

**Figure 2:**
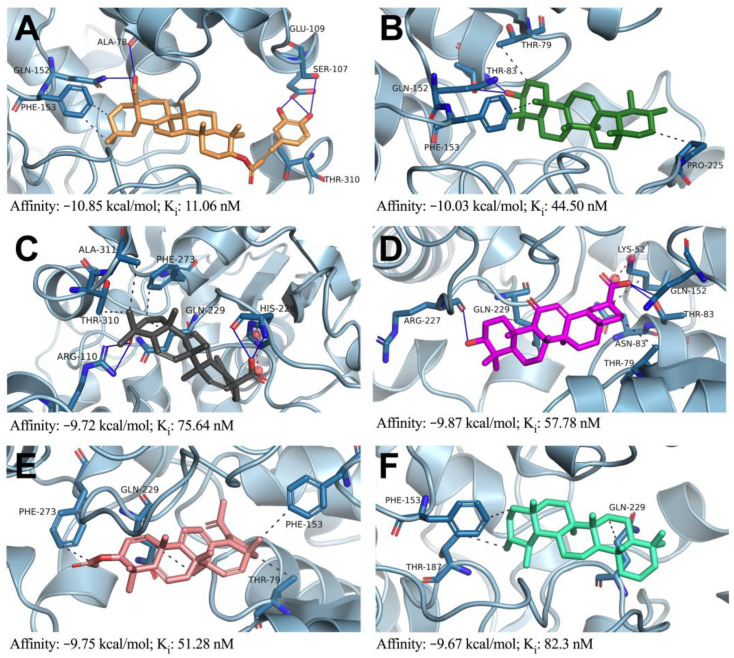
Lowest-energy docked pose of pentacyclic triterpenes with TcCS. (**A**) *3-O-cafeicoleanolic acid*. (**B**) α-Amyrin. (**C**) Ursolic acid. (**D**) Enoxolone. (**E**) Lupeol acetate. (**F**) Ursane. Interactions are represented as hydrogen bonds (blue lines), hydrophobic interactions (black dotted line), and ionic bonds (red dotted line).

The molecular interactions analysis showed that the pentacyclic triterpene ligands interacted most strongly with the TcCS enzyme, with values ranging from −9.2 to −10.3 kcal/mol. The compound with the best docking score, five random pentacyclic triterpenes and the reported inhibitor were subjected to redocking to analyze the types of interactions with the TcCS protein (Figure 2). The 3-O-cafeicoleanolic acid (ACAF) compound exhibited the best docking score and was shown to interact with six residues that conformed to the pocket in which the TcCS active site was located [29]. These interactions included five hydrogen bonds and three hydrophobic interactions (Figure 2A). The acid group hydroxyl of ACAF formed hydrogen bonds with the carbonyl group of the Ala78 residue and the amine group of Gln152. The remaining three hydrogen bonds were formed between the carbonyl and hydroxyl groups of Glu109 and Ser107 and the pyrocatechol of ACAF. Finally, the complex was stabilized by the formation of hydrophobic interactions with residues Phe153 and Thr310.

For the α amyrin (AMIR) compound, the hydroxyl group formed hydrogen bonds with residues Gln152 and Thr83 (Figure 2B). Residues Phe153, Thr79 and Pro225 were shown to be involved in hydrophobic interactions with the cyclic hydrocarbon part of the compound. The complex formed between the ursolic acid (AURS) compound and the TcCS enzyme was characterized by hydrogen bonds, hydrophobic interactions and ionic bond interactions (Figure 2C). Hydrogen bonds were formed between the hydroxyl groups of AURS, the guanidine group of Arg110, and the carbonyl and amine groups of His226. Hydrophobic interactions occurred between the carbonate backbone of AURS and the side chains of Ala311, Thr310, Gln229, and Phe273. Furthermore, the carboxylate group electrostatically interacted with the imidazole group of His226.

Similar interactions were observed for the TcCS and enoxolone (ENO) complex, where the carboxylic acid of the molecule formed an ionic bond with the amine of the Lys51 residue (Figure 2D). Likewise, in the TcCS–ENO complex, three hydrogen bonds formed between the hydroxyl groups of the molecule and the Arg227, Gln152, and Thr83 residues. Hydrophobic-type interactions formed between the ENO carbonate chain and the side chains of the Lys51, Thr79, Asn82, and Gln229 residues also contributed to the stability of the complex. It is important to note that the interactions of this ligand with the Lys51 and Asn82 residues have been reported to help stabilize the PLP cofactor in *L. major* [29]. The compounds ACLUPE and ursane (URS) are embedded in a hydrophobic pocket comprising residues Thr79, Phe153, Gln229, and Phe273 and Phe153, Thr187, and Gln229, respectively.

The energies of the interactions between the selected triterpenes and TcCS ranged from −9.67 to −10.85 kcal/mol. Based on the calculated interaction energies, the different triterpenes classified are as follows: ACAF > AMIR > ENO > ACLUPE > AURS > USR. The compounds ACAF (−10.85 kcal/mol) and AMIR (−10.03 kcal/mol) presented smaller docking energies than compound NSC61610 (−9.91 kcal/mol), which was used as a control. The inhibition constant (K_i_) of the triterpenes was directly proportional to the interaction energy, as expected. ACAF (11.06 nM), AMIR (44.5 nΜ) and ACLUPE (51.28 nM) exhibited lower concentrations than the inhibitor NSC61610 (54.15 nM), implying that they exhibited more TcCS inhibitory activity. Considering the docking energy and the inhibition constants, the ACAF, AMIR, and ACLUPE compounds were selected to evaluate complex stability via MD analyses.

### 3.4. Assessment of TcCS–Ligand Stability Using Molecular Dynamics Analysis

**Figure 3:**
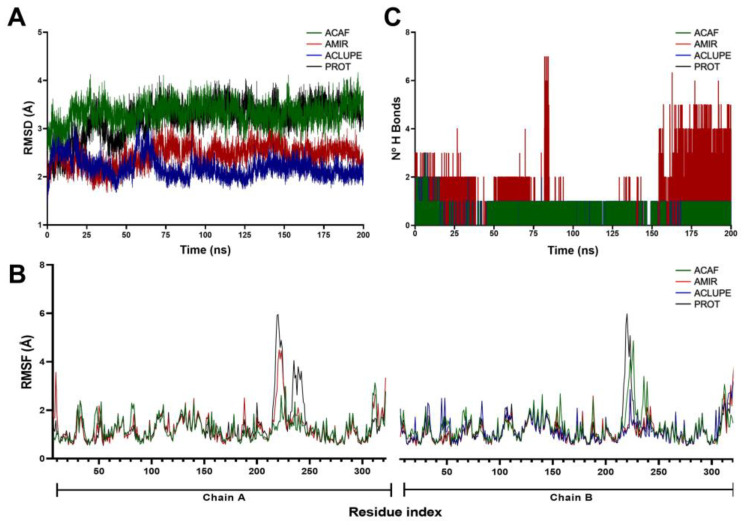
Molecular dynamics studies of potential triterpene inhibitors of the TcCS active site. (**A**) Root mean square deviation (RMSD). (**B**) Root mean square fluctuation (RMSF). (**C**) Hydrogen bonds between the TcCS protein and triterpene compounds throughout the simulations. The complexes of the cysteine synthase enzyme (PROT, black) with lupeol acetate (ACLUPE, blue), 3-O-cafeicoleanolic acid (ACAF, green) and α-amyrin (AMIR, red) complex are shown.

After docking studies, MD simulations of 200 ns were performed to characterize the stability over time of the interaction between TcCS and ACLUPE, AMIR, and ACAF compounds. First, the system stability was evaluated using the RMSD (root mean square deviation) value. In the RMSD of each system, it was observed that the ACLUPE and AMIR systems were the most stable during the 200 ns of MD. This supports that the systems remained stable during the simulation time (Figure 3A). In contrast, in the ACAF complex system, RMSD values gradually increase over the 200 ns of the simulation as evidenced by the low stability of the ACAF system.

The RMSF profiles of the complexes formed between TcCS and triterpenes revealed that the interactions with compounds exhibited low mobility in most of the residues, except for the regions surrounding the active site that comprised residues 200–250, in which the highest RMSF values were observed (Figure 3B). The greater flexibility of the residues adjacent to the active site formed between TcCS and the triterpenes may indicate a conformational change in the active site, which can lead to a decrease in the enzymatic catalytic activity. However, further analyses are required to corroborate this hypothesis.

In the analysis of hydrogen bonds during the 200 ns of simulation, it was observed that the TcCS–ACAF and TcCS–ACLUPE complexes are characterized by elapse dynamic time with the formation of a hydrogen bond. On the other hand, the TcCS–AMIR complexes pass between the times of 0 to 75 ns with two to three hydrogen bonds and between 150 and 200 ns with three to seven hydrogen bonds, thus showing a greater participation of hydrogen bonds in the TcCS–AMIR complex. Taking together molecular dynamics and molecular docking results, it is suggested that the stabilization of TcCS–triterpene complexes is mainly due to hydrophobic interactions and hydrogen bonds. The degree of relevance to each interaction, as well as the evaluation of cooperative effects of hydrogen bond–hydrophobic interaction were already described in other investigations [50], but should be explored in future studies in the TcCS–AMIR complex.

Based on the docking energies and interactions in the active site, as well as on the stability of the complexes over time, it was decided to delve into the ADMET properties and the trypanocidal capacities of the ACLUPE and AMIR compounds.

### 3.5. ADME and PAIN Predictions

The ADME and PAIN properties are important for determining potential toxicity and pharmacokinetic properties and for identifying potential false-positive structures (Table 4). The topological polar surface area (TPSA) is widely used as a molecular descriptor in the study of drug transport properties, such as intestinal absorption and penetration of the blood–brain barrier. The TPSA surface area is associated with heteroatoms, such as oxygen, nitrogen, and phosphorus and with polar hydrogen atoms. Compounds with poor absorption have been identified as those with a TPSA > 120 Å^2^ [51]. Herein, the compounds ACLUPE and AMIR showed good absorption properties based on its TPSA value of 26.3 and 20.23 Å^2^, respectively.

Typically, the most prevalent mechanism for drug absorption across cell membranes is passive diffusion, which requires drugs to possess enough lipophilicity to penetrate the lipid bilayer of cells. The lipophilicity of a drug can be expressed as the logarithm of its partition coefficient in an n-octanol/water system (Log Po/w) [52], where compounds with Po/w values > 5 are described as highly lipophilic. ACLUPE exhibited a Log Po/w of 7.67, a value similar to that found in AMIR Log Po/w of 7.05, demonstrating its ability to enter the cellular environment by passive diffusion [53]. However, these compounds showed high lipophilicity and therefore poor water solubility, which was also reflected by their estimated solubility (ESOL) [54].

Among the various routes of drug administration, the oral and cutaneous routes are preferred for patient comfort. Early oral estimates of bioavailability and skin penetration, that is, the fraction of the dose that reaches the bloodstream after oral administration and the ability to transport the drug through the skin, have been reported as key criteria for drug selection [55,56]. Mathematical models were used to predict the gastrointestinal (GI) absorption and skin penetration (LogKp) characteristics of the triterpenes molecules, revealing low gastrointestinal absorption and low skin penetration. Other important factors for understanding the pharmacokinetic characteristics of the selected compounds, such as blood–brain barrier permeance, P-gp substrate, and interaction with the isoforms of the P450 family of cytochromes (CYP1A2, CYP2C19, CYP2C9, CYP2D6, and CYP3A4), were negative, which suggests a low probability of inducing toxic adverse reactions or other unwanted effects [44].

Lipinski’s rules (LP.V) state that to be considered a good drug candidate, a molecule should have a molecular mass of less than 500 Da, no more than 5 hydrogen bond donors, no more than 10 hydrogen bond acceptors, and a LogPo/w lower than 5 [56]. The selected triterpenes failed to satisfy one of these rules, which was the LogPo/w value. However, it is important to clarify that Lipinski’s rules were initially designed to facilitate the development of drugs that are orally bioavailable, and although oral administration is a desirable goal for treating many tropical diseases, molecules that do not comply with all of these rules can still be explored by other experimental approaches for drug development.

Characterization of the suitability of the selected molecules for structural modification revealed the violation of two leadlikeness (LD.V) criteria, suggesting that these compounds are not suitable for structural modifications to improve its activity. Additionally, ACLUPE and AMIR exhibited a medium synthetic accessibility (SA) score, suggesting that its synthesis is moderately difficult; SA values have been reported to range from 1 (very easy) to 10 (very difficult) [44]. Finally, PAIN analysis to identify problematic structures within triterpenes compounds failed to reveal any interfering structures.

### 3.6. In Vitro Trypanocidal and Cytotoxic Activity

Considering the in silico analysis, the in vitro anti-*T. cruzi* and cytotoxic activities of ACLUPE and AMIR, it was observed that trypomastigote stage is more sensitive to ACLUPE compound than amastigote stage. ACLUPE induced death of trypomastigote with an EC_50_ of 15.82 ± 3.7 μg/mL and an inhibition of amastigote stage with an IC_50_ of 32.55 ± 1.2 μg/mL (Table 5). Furthermore, the AMIR compound showed the opposite behavior; the amastigote stage was more sensitive than trypomastigote stage. AMIR compound induced death and inhibition of *T. cruzi* during the trypomastigote and amastigote stages with an EC_50_ of 73.3 ± 1.85 μg/mL and IC_50_ of 9.08 ± 23.85 μg/mL, respectively. The trypanocidal effect was graded according to the EC_50_ or IC_50_ of each compound and classified as high potency (IC_50_ ≤ 10 μg/mL), moderate potency (IC_50_ = 10–20 μg/mL) and low/no activities (IC_50_ > 20 μg/mL) according to the method proposed by Isah et al. [42]. Thus, the ACLUPE compound was classified with moderate potency in trypomastigote stage, while the AMIR was classified with moderate potency in amastigote stage.

The cytotoxicity analysis of ACLUPE and AMIR compounds showed no toxic effect on VERO cells at the maximum concentrations evaluated, in fact, these compounds were less toxic than the reference compound NFX (Table 5).

The trypanocidal activity of ACLUPE, both as a component of extracts and as an isolated compound, have been documented and their mode of action have not yet been elucidated [57,58,59]. Petroleum ether extract obtained from *Kleinia odora* resulted in the elimination of *T. cruzi* at the trypomastigote stage, with a medium inhibitory concentration (IC_50_) of 5.7 ± 1.6 μg/mL, which is 3.4 times higher than that required to inhibit 50% of MRC-5 human fibroblast cells [57]. On the other hand, the ethyl acetate crude extract from *Cyrtocymura scorpioides*, exhibiting ACLUPE as the main component (41.15%), was demonstrated to inhibit *L. amazonensis* at the amastigote stage, with an IC_50_ of 16 ± 0.19 μg/mL and a selectivity index of 8.3 compared with the green monkey renal fibroblast-like cell model [58]. Moreover, the effects of the isolated compound on different trypanosome species have been evaluated, demonstrating its ability to inhibit promastigotes of *Leishmania* spp., with an IC_50_ of 30.0 µg/mL [59] and its ability to lyse 41.81 ± 5.14%, 78.80 ± 3.85%, and 79.40 ± 2.09% of *T. cruzi* trypomastigotes at concentrations of 100, 25, and 500 µg/mL, respectively [60]. In addition, natural-products lupeol isolated from aerial parts of *Vernonia scorpioides* also showed anti-trypanosomal activity with an IC_50_ of 12.48 µg/mL, but the mechanisms of action remain unexplored [61]. Others beneficial health effects of lupeol triterpenes have been documented, especially related to anti-inflammatory and anti-cancer effects [62,63]. These wide ranging pharmacological activities motive our current research interest on these compounds to be explored for the development of new therapeutic alternative strategy to control infectious diseases.

Regarding the trypanocidal capacities induced by AMIR, it has been found that extracts from *Eugenia pyriformis* leaves obtained using supercritical CO_2_ (E1) and ultrasound-assisted (E2), inhibit the epimastigote stage with an IC_50_ of 5.56 and 34.34 µg/mL, respectively. Likewise, they show lethal effects on the trypomastigote stage (E1 EC_50_: 16.69 µg/mL; E2 EC_50_: 7.80 µg/mL) without inhibiting mouse macrophages at the highest concentration used (300 µg/mL), reaching selectivity indices greater than 8.74 and 38.46, respectively. The characterization of the chemical components of the E1 extract found α-amyrin as the main component with 17.09 ± 0.27% of the relative abundance of the extract, a value similar to that found in the E2 extract where this compound represented 14.31 ± 0.36% [64].

The trypanocidal activity of AMIR in less complex mixtures or as an isolated compound is few and controversial. The evaluation of the α/β amyrin mixture in the trypomastigote stage did not observe a trypanocidal effect at the maximum concentration evaluated (100 μM). However, it was able to inhibit the amastigote stages with an IC_50_ of 20.2 ± 2.0 μM, suggesting a greater susceptibility of the amastigote stage compared to trypomastigote, a trend found in the present research. On the other hand, the assessment of the amyrin-isolated compounds has not been reported to inhibit the amastigote stage at a concentration less than 30 μM [65]. This contrasts with the findings of the present study where the IC_50_ values for AMIR were 24.84 ± 3.8 μM. However, the different response to treatment may be associated with the genetic diversity found in *T. cruzi*, which makes it necessary to confront the biological activities of the treating compounds in a variety of discrete typing units [66].

On the other hand, the variation in susceptibility between stages of the parasite may be due to multiple factors. Among these, is the influence of treatment on metabolic pathways associated with the biological function of the stage [67]. Other factors, such as differences in the environment of the parasites, levels and identity of the constituent metabolites of cell membranes [68], as well as the diffusion capacity and permeability of the constituent compounds of the extract in biological membranes, can also influence the differences in susceptibility between stages [69,70,71]. Considering that, the expression of cysteine synthase is different between trypomastigote and amastigote stages of the parasite [6], the difference in the response to ACLUPE and AMIR suggests that this type of compound can exert its trypanocidal effect through more than one mechanism.

## 4. Conclusions

The computational-based drug screening showed that terpenes, especially pentacyclic triterpenes, could occupy the pocket of the enzyme’s active site, thereby preventing the complexation of the pyridoxal phosphate cofactor and thus suggesting their ability to inhibit the catalytic activity of TcCS. Additionally, the assessment of the trypanocidal capacities of the selected compounds evidenced that the ACLUPE compound was selective and moderately potent in the trypomastigote stage, while AMIR was selective and moderately potent in the amastigote stage. Therefore, the present computational and experimental study allowed to identify promising pentacyclic triterpenes, especially ACLUPE and AMIR, to potentially inhibit *T. cruzi* cysteine synthase. These compounds may be explored as rational candidates for developing new therapeutic drugs for Chagas disease.

## Figures and Tables

**Table 1:** Selected terpenic and control compounds.

Compound	Chemical Formula	ZINC ID	PUBCHEM ID
Monoterpenes			
α-Pinene	C_10_H_16_	1530385	6654
α-Terpinene	C_10_H_16_	967594	7461
α-Thujene	C_10_H_16_	ND	17868
β-Pinene	C_10_H_16_	967582	14896
Camphor	C_10_H_16_O	967520	2537
Cineole	C_10_H_18_O	967566	2758
Limonene	C_10_H_16_	967513	22311
Perillic Acid	C_10_H_14_O_2_	1532418	1256
Tricyclene	C_10_H_16_	59778294	79035
Uroterpenol	C_10_H_18_O_2_	ND	93024
Sesquiterpenes			
α-Agarofuran	C_15_H_26_O	ND	10857022
α-Santalol	C_15_H_24_O	ND	5281531
α-Sinensal	C_15_H_22_O	4098303	5281534
Aromadendrene	C_15_H_24_	ND	91354
Artemisinin	C_15_H_18_O_4_	100014791	9838675
Bisabolene	C_15_H_24_	ND	3033866
Bisabolol	C_15_H_26_O	968461	1549992
Caryophylene Oxide	C_15_H_24_O	2039864	14350
Dehydroleucodin	C_15_H_16_O_3_	5202250	73440
Helenalin	C_15_H_18_O_4_	4098120	23205
Humulene	C_15_H_24_	38139375	5281520
Isoplumericin	C_15_H_14_O_6_	4098343	5281543
Mexicanin I	C_15_H_18_O_4_	4098149	93016
Nerolidol	C_15_H_26_O	1531550	5284507
Obtusol	C_15_H_23_Br_2_ClO	6018594	44583704
Peruvin	C_15_H_20_O_4_	64403867	52944698
Plumericin	C_15_H_14_O_6_	16343443	5281545
Polygodial	C_15_H_22_O_2_	4098293	72503
Psilostachyin A	C_15_H_20_O_5_	29134496	5320767
Spatulenol	C_15_H_24_O	13373002	92231
Diterpenes			
11-Hydroxysugiol	C_20_H_28_O_3_	ND	10403490
12-Hydroxydehydroabietic Acid	C_20_H_28_O_3_	ND	13370050
Abietic Acid	C_20_H_30_O_2_	2267806	10569
Communic Acid	C_20_H_30_O_2_	ND	637125
Dolabelladienetriol	C_20_H_34_O_3_	ND	6477027
Eleganolone	C_20_H_32_O_2_	14654612	6439034
Geranylgeraniol	C_20_H_34_O	1531391	5281365
Kaurenoic acid	C_20_H_30_O_2_	101485632	73062
Lambertic Acid	C_20_H_28_O_3_	4720783	13370049
Salviol	C_20_H_30_O_2_	ND	13966146
Sugikurojin A	C_20_H_28_O_2_	ND	12116652
Serterterpene			
Drimanial	C_25_H_30_O_6_	ND	636975
Tetracyclic Triterpenes			
Cycloartenol	C_30_H_50_O	118937272	500213
Dammarenediol	C_30_H_52_O_2_	ND	10895555
Lanosterol	C_30_H_50_O	3870056	246983
Pentacyclic Triterpenes			
20 α-Hydroxytingenone	C_28_H_36_O_4_	66100553	10717799
28-Hydroxyisoiguesterin	C_28_H_36_O_3_	ND	10622044
3-O-Caffeicoleanolic Acid	C_39_H_54_O_6_	ND	24873431
3-O-Trans-Caffeoyltormentic Acid	C_39_H_54_O_8_	ND	44584640
α-Amyrin	C_30_H_50_O	100780293	73170
Alisol B	C_30_H_48_O_4_	26828734	15558620
Asiatic acid	C_30_H_48_O_5_	8221271	119034
β-Amyrin	C_30_H_50_O	3978270	73145
Betulinic Acid	C_30_H_48_O_3_	118937400	64971
Boswellic acid	C_30_H_48_O_3_	14089743	168928
Celastrol	C_29_H_38_O_4_	19795938	122724
Enoxolone	C_30_H_46_O_4_	19203131	10114
Epikatonic Acid	C_30_H_48_O_3_	32296198	636467
Euscaphic Acid	C_30_H_48_O_5_	43552893	471426
Hydroxygeningenone	C_28_H_36_O_4_	ND	500289
Isoiguesterin	C_28_H_36_O_2_	31761332	11373102
Lupane	C_30_H_52_O_2_	14720190	9548715
Lupeol Acetate	C_32_H_52_O_2_	ND	92157
Murrayenol	C_30_H_46_O_4_	ND	73086673
Myrianthic Acid	C_30_H_48_O_6_	ND	14055735
Oleanolic Acid	C_30_H_48_O_3_	3785416	10494
Pomonic Acid	C_30_H_46_O_4_	15118611	12314449
Pristimerin	C_30_H_40_O_4_	4097723	159516
Pubesenolide	C_28_H_42_O_5_	118936969	44249449
Sumaresinol	C_30_H_48_O_4_	4349900	12443148
Ursane	C_30_H_52_	ND	9548870
Ursolic Acid	C_30_H_48_O_3_	3978827	64945
Terpenic Coumarins			
2-Epi-Helmanticine	C_26_H_34_O_10_	ND	102272642
Auraptene	C_19_H_22_O_3_	1658901	1550607
Farnesiferol B	C_24_H_30_O_4_	29134692	1779468
Farnesiferol C	C_24_H_30_O_4_	28107226	15559239
Galbanic Acid	C_24_H_30_O_5_	4042371	7082474
Methyl Galbanate	C_25_H_32_O_5_	4025386	7075765
Szowitsiacoumarin A	C_24_H_30_O_4_	ND	102272640
Szowitsiacoumarin B	C_24_H_30_O_5_	ND	102272641
Umbelliprenin	C_24_H_30_O_3_	2126785	1781413
Controls			
NSC61610	C_34_H_24_N_6_O_2_	ND	247228
O-Acetyl-DL-Serine	C_5_H_9_NO_4_	ND	189
Pyridoxal Phosphate	C_8_H_10_NO_6_P	1532514	1051

ND: Not determined.

**Table 2:** Molecular docking energies of the interactions between the terpenic compounds and CS enzymes.

Compound	TcCS	LmCS	Compound	TcCS	LmCS
	(kcal/mol)			(kcal/mol)	
Monoterpenes (n = 10)			Pentacyclic Triterpenes (n = 27)		
α-Pinene	−5.3	−5.5	20-α-Hydroxytingenone	−10.1	−10.7
α-Terpinene	−5.2	−6.2	28-Hydroxyisoiguesterin	−9.6	−10.7
α-Thujene	−5.2	−5.4	3-O-Caffeicoleanolic Acid	−10.3	−12.2
β-Pinene	−5.3	−5.5	3-O-Trans-Caffeoyltormentic Acid	−10.0	−11.5
Camphor	−5.4	−5.6	α-Amyrin	−9.8	−10.1
Cineole	−5.6	−5.8	Alisol B	−9.6	−10.4
Limonene	−5.2	−5.9	Asiatic acid	−9.6	−10.4
Perillic Acid	−6.7	−6.4	β-Amyrin	−9.7	−11.0
Tricyclene	−4.9	−5.9	Betulinic Acid	−9.5	−10.0
Uroterpenol	−5.8	−6.2	Boswellic acid	−9.6	−11.0
Sesquiterpenes (n = 20)			Celastrol	−9.8	−10.7
α-Agarofuran	−6.7	−6.9	Enoxolone	−10.1	−11.0
α-Santalol	−5.8	−7	Epikatonic Acid	−9.8	−10.7
α-Sinensal	−5.9	−6.3	Euscaphic Acid	−9.4	−10.1
Aromadendrene	−6.4	−6.4	Hydroxygeningenone	−9.8	−10.7
Artemisinin	−7.6	−8.8	Isoiguesterin	−9.8	−10.8
Bisabolene	−5.4	−7.4	Lupane	−9.2	−10.2
Bisabolol	−6.0	−6.8	Lupeol Acetate	−9.4	−10
Caryophylene Oxide	−6.3	−7.1	Murrayenol	−9.2	−10.7
Dehydroleucodin	−7.1	−8.2	Myrianthic Acid	−9.6	−10.3
Helenalin	−7.3	−8.6	Oleanolic Acid	−9.5	−11.2
Humulene	−6.5	−6.6	Pomonic Acid	−9.2	−11.5
Isoplumericin	−7.4	−8.8	Pristimerin	−9.2	−10.2
Mexicanin I	−7.6	−8.7	Pubesenolide	−9.2	−11.4
Nerolidol	−5.2	−6.3	Sumaresinol	−9.6	−11.2
Obtusol	−7.0	−7.5	Ursane	−9.5	−10.7
Peruvin	−7.5	−8.2	Ursolic Acid	−9.6	−10.7
Plumericin	−7.5	−8.9	Terpenic Coumarins (n = 9)		
Polygodial	−6.2	−6.9	2-Epi-Helmanticine	−7.5	−10
Psilostachyin A	−8.4	−9.0	Auraptene	−6.4	−8.6
Spatulenol	−6.7	−7.1	Farnesiferol B	−8.3	−10.2
Diterpenes (n = 11)			Farnesiferol C	-8.5	-10.0
11-Hydroxysugiol	−7.9	−9.0	Galbanic Acid	−7.4	−9.9
12-Hydroxydehydroabietic Acid	−8.5	−9.0	Methyl Galbanate	−7.5	−10.0
Abietic Acid	−7.3	−7.9	Szowitsiacoumarin A	−8.9	−10.7
Communic Acid	−7.1	−7.7	Szowitsiacoumarin B	−9.0	−10.7
Dolabelladienetriol	−8.1	−9.1	Umbelliprenin	−6.8	−9.3
Eleganolone	−6.8	−7.9	Controls (n = 3)		
Geranylgeraniol	−6.2	−7.1	NSC61610	−9.6	−12.6
Kaurenoic acid	−8.0	−8.7	O-Acetyl-DL-Serine	−5.6	−5.7
Lambertic Acid	−8.2	−8.7	Pyridoxal Phosphate	−6.6	−7.5
Salviol	−7.8	−8.2			
Sugikurojin A	−8.0	−7.1			
Serterterpene (n = 1)					
Drimanial	−7.4	−8.9			
Tetracyclic Triterpenes (n = 3)					
Cycloartenol	−8.4	−9.4			
Dammarenediol	−8.8	−10.5			
Lanosterol	−8.3	−9.3			

**Table 3:** Mean molecular docking energies of the interactions of the different types of terpenic compounds with CS enzymes.

	TcCS		LmCS	
Compound Type	MDE (kcal/mol)	% RSD	MDE (kcal/mol)	% RSD
Monoterpenes (n = 10)	−5.46	9.15	−5.84	5.89
Sesquiterpenes (n = 20)	−6.73	12.44	−7.58	12.78
Diterpenes (n = 11)	−7.63	9.09	−8.22	8.97
Sesterterpenes (n = 1)	−7.4	--	−8.9	--
Tetracyclic Triterpenes (n = 3)	−8.50	3.11	−9.73	6.84
Pentacyclic Triterpenes (n = 27)	−9.62	3.07	−10.74	4.93
Terpenic Coumarins (n = 9)	−7.81	11.71	−9.93	6.60

MDE—Mean docking energy, % RSD—relative standard deviation percentage of docking energy.

**Table 4:** Calculated physicochemical properties and ADME profile of the lupeol acetate and α-amyrin compounds.

Compound	MW	HBA	HBD	TPSA	cLogPo/w	ESOL logs	PAINs
Lupeol acetate	458.675	2	0	26.3	7.67	−9.13	0
	ESOL class	GI abs.	LogKp (SP)	LP.V	LD.V	SA	
	Poorly soluble	low	−1.74	1	2	5.66	
Compound	MW	HBA	HBD	TPSA	cLogPo/w	ESOL logs	PAINs
α-amyrin	426.72	1	1	20.23	7.05	−8.16	0
	ESOL class	GI abs.	LogKp (SP)	LP.V	LD.V	SA	
	Poorly soluble	low	−2.51	1	2	3.17	

MW—Molecular weight; HBA—hydrogen bond acceptor; HBD—hydrogen bond donor; TPSA—topological polar surface area; cLogPo/w—octanol/water partition logarithm consensus; ESOL—estimated solubility logarithm; GI abs—gastrointestinal absorption; SP—skin penetration; LP.V—Lipinski’s rule violation; LD.V—leadlikeness violation; SA—synthetic accessibility; PAINs—pan-assay interference compounds.

**Table 5:** Activity of ACLUPE and AMIR compounds against Trypanosoma cruzi and cytotoxic effects assessment.

Compound	μg/mL			SI	
	EC_50_ TRY	IC_50_ AMA	CC_50_ VERO	TRY	AMA
ACLUPE	15.82 ± 3.7	32.55 ± 1.2	>94	>5.94	>2.88
AMIR	73.3 ± 1.85	9.08 ± 2.5	>85	>1.15	>9.36
NFX	2.49 ± 1.80	3.07 ± 0.96	13.7 ± 6.23	5.50	4.46

EC_50_—Half maximal effective concentration, IC_50_—half maximal inhibitory concentration, CC50—Half maximal cytotoxicity concentrations, SI—Selectivity Index, TRY—Trypomastigotes, AMA—Amastigotes, ACLUPE—Lupeol acetate, AMIR—α-amyrin, NFX—Nifurtimox.

## Data Availability

All data generated or analyzed are available in this published article and its supplementary materials.

## Supplementary Materials

The following supporting information can be downloaded at: https://www.mdpi.com/article/10.3390/tropicalmed8050263/s1, Figure S1: Stereochemical quality model for TcCS enzyme; Figure S2: Stereochemical quality model for LmCS enzyme.
