# Supplementary figures and images for: Lupeol Acetate and α-Amyrin Terpenes Activity against Trypanosoma cruzi: Insights into Toxicity and Potential Mechanisms of Action
Source: Trop Med Infect Dis. 2023 May 3;8(5):263. doi: 10.3390/tropicalmed8050263 (PMC10220761; doi:10.3390/tropicalmed8050263)

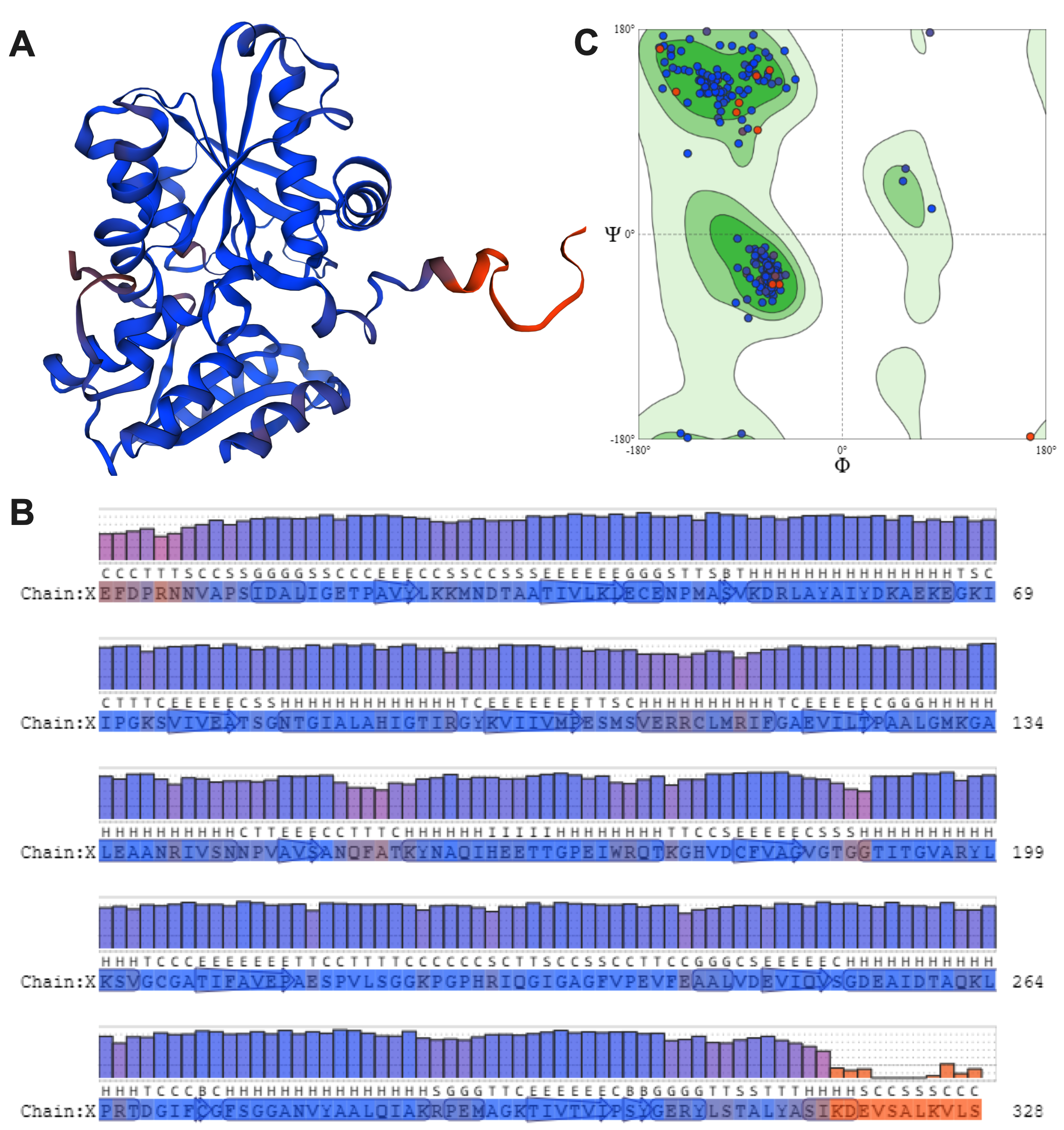

Supplement: Supplementary file 1 [file tropicalmed-08-00263-s001.zip › Figure S1 .tiff]

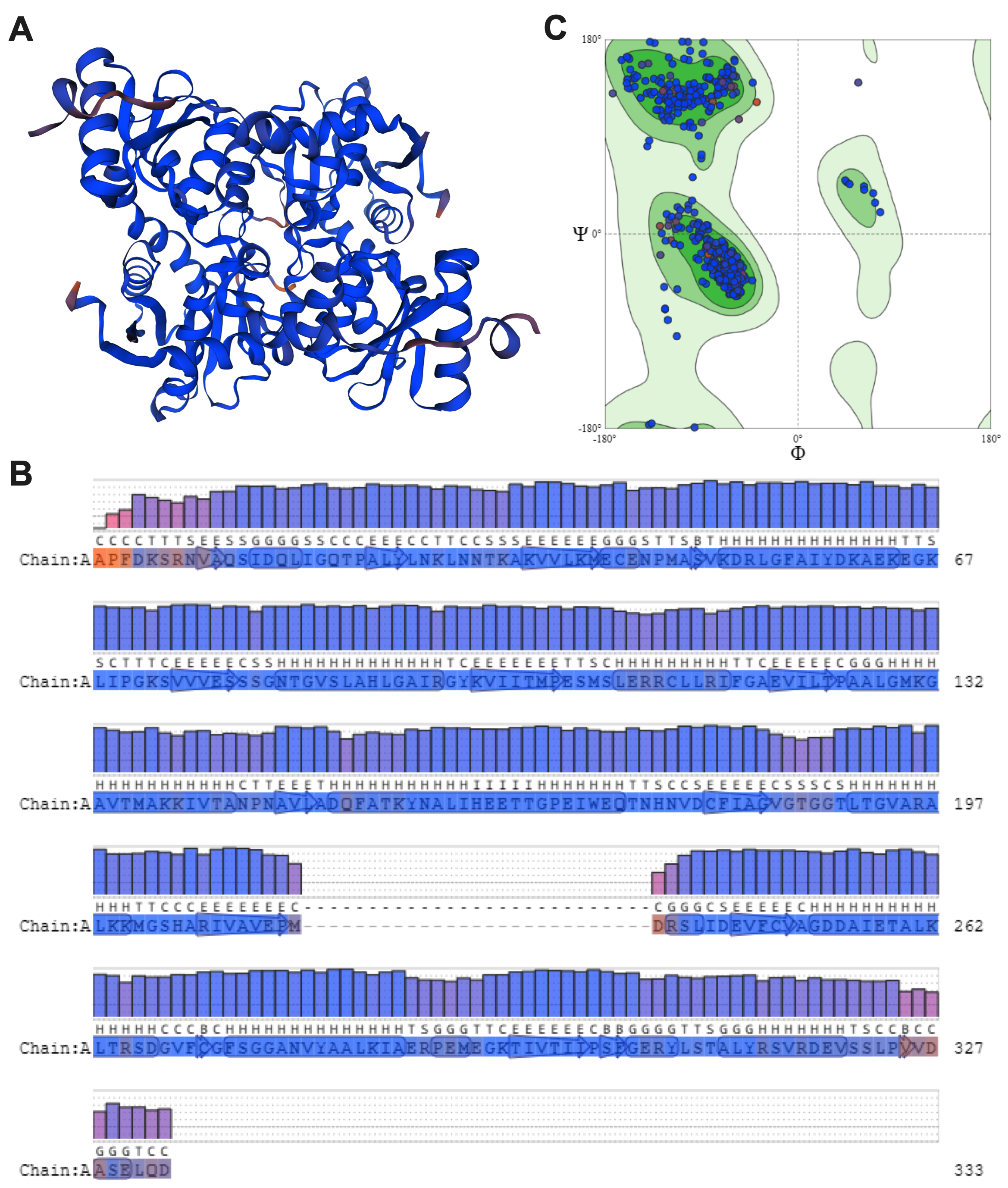

Supplement: Supplementary file 1 [file tropicalmed-08-00263-s001.zip › Figure S2 .tiff]
